# The clinical characteristics and surgical strategy in infants with lingual thyroglossal duct cyst

**DOI:** 10.1016/j.bjorl.2024.101536

**Published:** 2025-01-03

**Authors:** Fenghua Qin, Yihua Ni, Wenxia Chen

**Affiliations:** Children’s Hospital of Fudan University, Department of Otolaryngology-Head and Neck Surgery, Shanghai, China

**Keywords:** Lingual thyroglossal duct cyst, Infants, Laryngomalacia

## Abstract

**Objectives:**

Lingual Thyroglossal Duct Cysts (LTDCs) are a rare variant of thyroglossal duct Cyst (LDC). This study aimed to explore the efficacy of transoral excision of LTDC and evaluate the added benefit of concomitant management of laryngomalacia during the surgical intervention.

**Methods:**

Infants with LTDCs were retrospectively collected from our department from January 2009 to January 2022. The baseline characteristics, clinical symptoms, the features of transnasal flexible fiberoptic laryngoscopy, CT and MRI, surgical treatments, and follow-up results were described and analyzed.

**Results:**

50 babies with LTDCs were included. Five infants had urgent bedside cyst aspiration prior to surgery for respiratory distress. All patients underwent cyst resection. Concurrent LTDC and laryngomalacia were found in 10 patients on preoperative laryngoscopy. Due to breathing and swallowing issues, these infants underwent transoral cyst removal and supraglottoplasty in the same surgical setting. 3 large cyst cases, laryngomalacia were undetectable pre-cystectomy. When extubation failed after 1weeks, repeat laryngoscopy revealed severe laryngomalacia necessitating successful supraglottoplasty. All patients were discharged from the hospital once oral intake is safe and adequate.

**Conclusion:**

Transoral surgery for LTDC is an effective and safe surgical treatment. Additionally, identifying and promptly addressing other laryngeal conditions, such as laryngomalacia, significantly enhances the success rate of the surgery.

**Level of evidence:**

4.

## Highlights

• A total of 50 babies with LTDCs underwent cyst resection.
• 10 patients underwent simultaneous cystectomy and supraglottoplasty.
• 3 cysts underwent supraglottoplasty due to extubation failure after cystectomy.
• Rigorous post-operative laryngoscopy can identify hidden laryngeal malformations.

## Introduction

Thyroglossal Duct Cyst (TDC) is the most common embryonic-origin cervical mass discovered in the anterior neck, accounting for 75% of congenital neck abnormalities.^1^ Residual thyrolingual duct in the tongue body can form a lingual TDC. LTDC is rare, comprising just 0.6%–3% of all TDCs.^2^ The majority of LTDCs occur in infants.^3^ Compared to TDC at other locations, LTDC is more frequently symptomatic, often presenting as a prominent tongue base mass with respiratory distress or even fatal upper airway obstruction, especially in early infancy.^4^

Patients with LTDC are often overlooked or misdiagnosed with laryngomalacia, the most common cause of pediatric stridor and a frequent referral to pediatric otolaryngologists.^5^, ^6^ Flexible laryngoscopy is the primary method of examination and key diagnostic tool for LTDC and laryngomalacia,^6^ while imaging (CT/MRI) and thyroid scintigraphy are crucial for the differential diagnosis of LTDC.

Endoscopic or microscopic low-temperature coblation resection has achieved good therapeutic outcomes for LTDC.^7^, ^8^

This study reports on the transoral excision of 50 LTDCs, where laryngomalacia identified during preoperative laryngoscopy was addressed synchronously during surgery. Postoperative follow-up laryngoscopy revealed previously undiagnosed laryngomalacia affecting respiration, which was subsequently managed surgically.

## Methods

Patients with cysts present only at the tongue base were selected for review from all patients who underwent surgical excision of tongue base lesions at Children's Hospital of Fudan University, from January 2009 to January 2022. These patients came primarily from the departments of neonatology, NICU, PICU and ENT. Patients who had cystectomy localized only to the tongue base were selected from this group for specific review. Clinic charts, radiographic images, laryngoscopy reports, and operative procedures were examined. Information was gathered and compiled into a central database.

The onset of symptoms ranged from 1 day to 311 days (average: 29-days). Ten children had symptoms present at birth, while 33 developed gradually within 3 weeks. 4 patients (8%) were found incidentally through imaging or laryngoscopy. 30 patients (60%) presented with breathing issues without severe desaturation. 3 patients (6%) required intubation for airway protection. 7 patients (14%) had a history of apnea/cyanosis, and 3 others (6%) had obstructive sleep apnea and failure to thrive. 3 older patients (6%) had only mild tongue discomfort and odynophagia. Baseline characteristics of the 50 patients are described in Table 1.

**Table 1:** Baseline characteristics and clinical symptoms of LTDC infants (n = 50).

Characteristics	Total
Age, average (IQR)	
Presentation of symptoms	29 (1−311) days
Diagnosis with endoscopy	32 (1−328) days
Surgery	49 (3−333) days
Sex, n (%)	
Male	32 (64.0)
Female	18 (36.0)
Gestational age, mean (SD)	35.8 (2.1) weeks
Birth weight, mean (SD)	3258 (386.2) g
Clinical symptoms, n (%)	
Mild breathing issues	30 (60)
Intubation	3 (6)
Sever apnea/cyanosis	7 (14)
Snore & Feeding problems	3 (6.0)
Mild tongue discomfort and odynophagia	3 (6.0)
No symptoms n (%)	4 (8)
Type of laryngomalacia, n	
Type I	3
Type II	5
Type III	1
Type I + II	3
Type I + III	1
Surgical treatments, n (%)	
Cystectomy	50 (100.0)
Supraglottoplasty	13 (26)

Patients were excluded if clinical documentation (including clinical notes, operative reports, imaging and laryngoscopy reports) was missing or inconsistent, or if the follow-up period was less than 12 months.

**Fig. 1:**
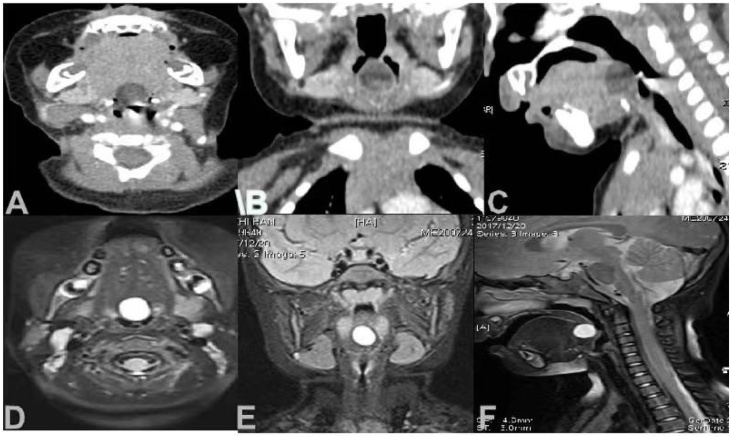
(A, B, C) CT of larynx. A round cyst of low density can be seen at the base of the tongue. (D, E, F) MRI of larynx. A round cyst can be seen at the base of the tongue.

Laryngoscope, laryngeal CT and MRI are important for the diagnosis of LTDC (Fig. 1). Radiologically, the cyst's close proximity to the hyoid bone is the most valuable imaging feature of LTDC. The measurable cysts were similar in size, with the smallest being 0.6 cm in diameter and the largest 2.5 cm in greatest dimension (average: 1.6 cm). Along with routine preoperative electronic nasopharyngolaryngoscopy, we were able to exclude choanal atresia and other upper airway congenital malformations.

This work was approved by the Ethics Committee of Fudan University.

### Surgical technique

**Fig. 2:**
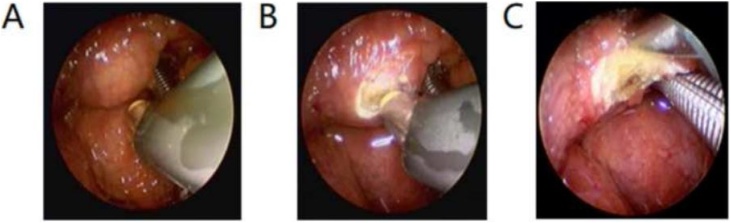
(A) Image depicting the laryngoscopy of LTDC prior to the removal of cysts. (B) Low temperature radiofrequency ablation surgery. (C) Final appearance of the cavity after the removal of cysts.

**Fig. 3:**
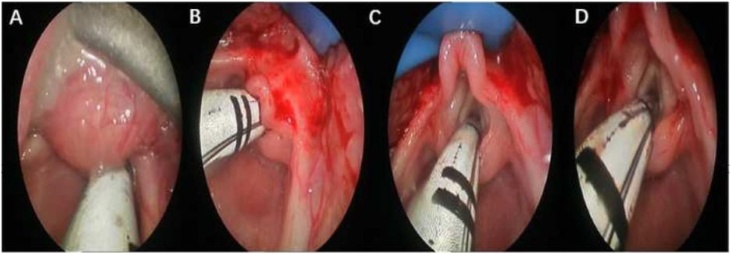
(A) Image depicting laryngoscopy of an LTDC prior to cyst removal. (B) Intraoperative photo after cyst removal with Straightshot™ M5 microdebrider powered by multispecialty Integrated Power Console (IPC™) system. (C) After cyst removal, the position of the supporting laryngoscope was adjusted, revealing type II laryngomalacia, shortened aryepiglottic folds associated with a long, omega-shape epiglottis that curls on itself. (D) The aryepiglottic folds after being trimmed by laryngeal scissors.

**Fig. 4:**
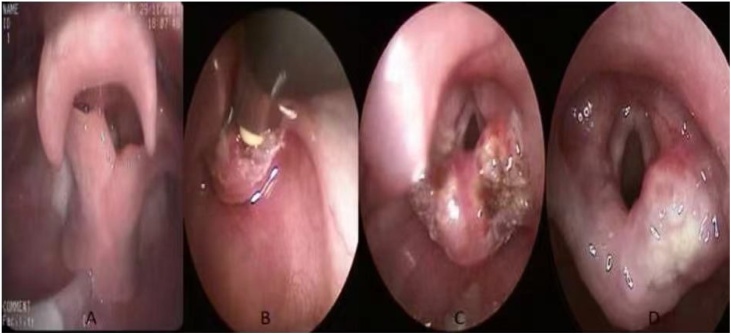
One patient diagnosed with type I laryngomalacia required supraglottoplasty with low temperature radiofrequency ablation after cyst removal alone failed to improve breathing.

After successful nasotracheal intubation under general anesthesia, the cyst was exposed using a laryngoscope. The entire tongue base, vallecula and epiglottis were visualized by positioning the laryngoscope. Applying pressure around the lesion with the laryngoscope along the tongue base helped display its borders. Throat forceps were used to remove LTDC tissue for histopathological examination. Low-temperature radiofrequency ablation (Fig. 2) or the Straightshot TM M5 microdebrider powered by the Multispecialty Integrated Power Console (IPCTM) system (Fig. 3) were used to achieve hemostasis and completely ablate or cut the entire surface of the resection cavity. This ensured complete ablation of all microscopic mucosal tissue to prevent recurrence. The resection cavity was left open to heal to decrease the risk of burying mucosalized tissue with closure. After cyst excision, 10 patients with laryngomalacia diagnosed preoperatively had supraglottoplasty performed, with the laryngoscope position adjusted to adequately expose the epiglottis, aryepiglottic folds, and vocal cords according to the type of laryngomalacia (Fig. 3). 3 patients required supraglottoplasty within 1week after cyst removal alone failed to improve breathing (Fig. 4). Five infants had urgent bedside cyst aspiration prior to surgery for respiratory distress, with 3 done at NICU and 2 at outside institutions.

## Results

From 2009 until 2022, 55 operations were performed on 50 patients with tongue base cysts, including 32 boys and 18 girls. All 50 patients underwent transoral cyst excision. The average age at surgery was 49 days (range: 3–333 days).

### Postoperative course

Postoperatively, 45 (90%) patients remained intubated overnight in the PICU or NICU to prevent surgical edema from affecting ventilation, achieve better epiglottic positioning, and avoid reagglutination of the aryepiglottic folds after supraglottoplasty. 5 patients had a history of extubation. 3 were reintubated that night due to respiratory instability, while 2 with stable breathing were not reintubated. 5 patients with smaller cysts (<1.0 cm) and less surgical edema were extubated after 40 min to 1 h of observation in the recovery room and then transferred to the general ward. 38 patients were successfully extubated on postoperative day 1, while 5 failed extubation at 24 h. 2 required re-operation for postoperative hemorrhage leading to worsened edema and delayed extubation. 45 were discharged from the hospital on postoperative days 2‒3. The remaining 5 patients had persistent stridor and labored breathing causing extubation failure and longer hospital stays. 3 patients ultimately underwent supraglottoplasty for laryngomalacia (type II in 1, type I + II in 2) within 1 week after cyst removal, resolving their symptoms. The other 2 patients with stridor and labored breathing were found to have preoperative pneumonia that worsened after surgery, requiring infliximab nebulizer treatment. These patients were discharged after improvement. All patients were discharged from the hospital once oral intake was safe and adequate.

### Pathology

Pathology reports were available. The specimen was consistent with thyroglossal duct cyst, confirmed by the presence of respiratory epithelium, columnar epithelium, or squamous epithelium.

### Follow-up

**Fig. 5:**
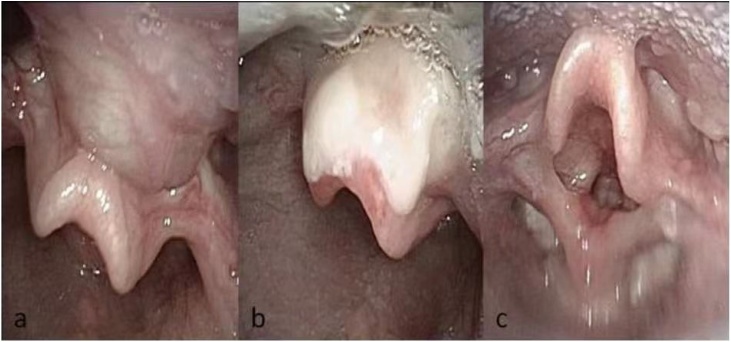
Laryngoscopic photos in outpatient visits. (a) Laryngoscopy reveals a cyst at an outpatient visit prior to surgery. (b) Laryngoscopy 1 week after surgery at an outpatient visit. (c) Laryngoscopy 4 weeks after surgery at an outpatient visit.

The follow-up period was defined as the time from surgery to the most recent clinical evaluation. The average follow-up length was 1.5 years, ranging from 1 to 2 years. Flexible fiberoptic laryngoscopy was mostly performed at 1 week, 4 weeks, 3 months, and 6 months postoperatively to evaluate the surgical site and to ensure that there was no recurrence of the cyst. The base of tongue healed very well after surgery (Fig. 5). Breathing improved greatly in nearly all patients. Most had only mild transient side effects like odynophagia and dysphagia. In summary, the vast majority of patients had resolution of respiratory symptoms and good outcomes after surgery, with only minor transient side effects. No recurrences were observed over the follow-up period.

## Discussion

In the neonatal period and up to 6 months after birth, the hypopharyngeal cavity is narrow and has low resistance. Large cysts can protrude into the anterior part of the tongue, causing obstructive symptoms.^9^ Pediatricians should perform flexible laryngoscopy on infants presenting with laryngeal stridor, respiratory disorders, deglutition difficulties, or sleep-related symptoms to assess for upper airway obstruction.^10^ Diagnosing LTDC can be more challenging compared to typical TDC.

Laryngeal CT and MRI are crucial for diagnosing LTDC that do not fully protrude into the laryngopharynx and can be missed during laryngoscopy.^11^, ^12^, ^13^, ^14^ Preoperative laryngoscopy not only helps detecting LTDC but also identifies other laryngeal abnormalities, such as congenital laryngomalacia.^5^, ^6^

If LTDCs significantly enlarge, they can obstruct breathing and swallowing. In urgent cases, puncturing and draining the cyst using a laryngoscope may be necessary. In our study, we have 5 patients underwent puncturing before surgery. However, this procedure carries risks of bleeding and infection, so it is usually reserved for emergency situations. Long-term management of LTDC involves surgical removal to prevent recurrence. The Sistrunk procedure, including limited tongue base excision and suture-guided transhyoid pharyngotomy, is a recommended strategy for TDCs.^15^, ^16^ However, traditional open Sistrunk procedures or tongue base dissections can lead to significant morbidity, especially in infants, due to the risk of wound complications, hemorrhage, infection, and respiratory edema. Transoral approaches aim to reduce these risks by minimizing overall morbidity and avoiding extensive dissections of the neck, tongue base, hyoid, and suprahyoid regions.

In our study, among the 50 pediatric patients with cysts, 10 cases were removed by Straightshot TM M5 microdebrider and 40 cases by plasma ablation^7^, ^23^ by transoral approaches, based on the preferences and habits of different surgeons. There was no recurrence after surgery in all cases.

Burkart et al. reported positive outcomes with no complications or recurrences in 16 cases with endoscopic transoral excision for LTDCs, despite prolonged intubation.^17^ In contrast, Zhang et al. reported that recurrence was observed following transoral endoscopic cystectomy or marsupialization in seven patients with LTDC.^18^ Sameer recommended transoral marsupialization for lingual TDC in infant patients due to its low morbidity and high success rate.^19^ Fong et al. have demonstrated the feasibility of transoral endoscopic excision for LTDC with no significant recurrence rates.^20^

Recently, successful transoral robotic resection of LTDC has been performed in adults^21^ and infants.^22^ Previously, there were alternative procedures for ingrowth TDC (e.g., marsupialization or endoscopic complete excision), which may have been more effective than Sistrunk surgery in treating infants with LTDC.^21^, ^22^

Laryngomalacia can lead to stridor, difficulty feeding, apnea, and cyanosis. For 10 cases with small cysts (<0.9 cm), laryngoscopic examination prior to surgery revealed concurrent severe laryngomalacia. The diagnosis of laryngomalacia is confirmed with flexible fiberoptic laryngoscopy based on one or a combination of three primary findings: arytenoid prolapse, foreshortened aryepiglottic folds, and a posteriorly displaced omega-shaped epiglottis. After cyst removal, supraglottoplasty was performed by adjusting the laryngoscope position. For patients with large cysts, the conditions of the epiglottis, aryepiglottic folds, and vocal cords could not be assessed preoperatively, making it impossible to determine if laryngomalacia was present. Routine postoperative electronic laryngoscopic follow-ups identified 3 cases with severe laryngomalacia who failed routine extubation 1 week after cyst surgery. These patients subsequently underwent supraglottoplasty, which enabled successful extubation. Studies^24^ have shown that laryngomalacia typically resolves over months without supraglottoplasty. However, complete symptomatic improvement with supraglottoplasty occurs within weeks. Supraglottoplasty is also a surgery with high efficacy and is associated with low morbidity.^25^

The removal of the cyst with supraglottoplasty was able to achieve a better stable airway,^26^ which was consistent with the results of our study. This also shows the critical importance of periodic postoperative laryngoscopic follow-up examinations. After surgery, the base of tongue healed very well. Breathing improved greatly in nearly all patients. Improvements were also found in the overall health, physical ability, and satisfaction with growth and development of all patients. As a result, there was no recurrence, and none of our cases had any postoperative problems during the follow-up period. Our procedure is necessary and sufficient.

This study provides compelling findings based on a thorough review of a large patient group, although causative mechanisms remain incompletely defined. Ongoing studies using a prospective, analytically rigorous methodology focused on histopathologic factors may elucidate the pathogenesis of LTDC. Despite the need for continued research, this study significantly advances current understanding of diagnosis and management for this rare entity. The relatively short follow-up time is one limitation of this paper, and continuous follow-up is required.

## Conclusions

In summary, optimal outcomes require thorough preoperative diagnosis using imaging, laryngoscopy, and symptom evaluation; precise cyst removal with concurrent treatment of laryngeal disease; and vigilant postoperative laryngoscopic surveillance to identify any residual or new conditions needing further treatment. This systematic approach facilitates successful management of complex LTDC cases throughout all perioperative stages.

## Funding

No.

## Declaration of competing interest

No conflict of interest exits in the submission of this manuscript, and manuscript is approved by all authors for publication.
